# Technologically advanced running shoes reduce oxygen cost and cumulative tibial loading per kilometer in recreational female and male runners

**DOI:** 10.1038/s41598-024-62263-0

**Published:** 2024-05-24

**Authors:** Amelie Werkhausen, Magne Lund-Hansen, Lucas Wiedenbruch, Klaus Peikenkamp, Hannah Rice

**Affiliations:** 1 Department of Physical Performance, Norwegian School of Sport Sciences, Sognsveien, 220, 0863 Oslo, Norway; 2 Section for Pharmacy, Intelligent Health Initiative, Department of Life Sciences and Health, Oslo Metropolitan University, Oslo, Norway; 3 Department of Engineering Physics, FH Münster University of Applied Sciences, Münster, Germany

**Keywords:** Running biomechanics, Running footwear, Supershoes, Tibial loading, Physiology, Bone

## Abstract

Technologically advanced running shoes (TARS) improve performance compared to classical running shoes (CRS). Improved race performance has been attributed to metabolic savings in male runners, but it remains unclear if these same benefits are experienced among females and in recreational runners. The mechanisms behind these benefits are still not fully understood despite the need for optimisation, and their influence on injury mechanisms has not been explored. Here we combined biomechanical, physiological, and modelling approaches to analyse joint mechanics, oxygen uptake, and tibial load in nineteen male and female recreational runners running with CRS and TARS at their individual lactate threshold speed (12.4 ± 1.9 km/h). Oxygen uptake was 3.0 ± 1.5% lower in TARS than in CRS. Ankle dorsiflexion, joint moment and joint power were reduced in TARS compared to CRS at various phases of stance including midstance, while knee joint mechanics were mostly similar throughout. There were no significant differences for tibial bending moment during the stance phase but cumulative tibial damage per kilometre was 12 ± 9% lower in TARS compared to CRS. Our results suggest that running with TARS reduces oxygen cost in recreational female and male runners, which may partly be explained by differences in lower limb joint mechanics. The lower cumulative tibial bone load with TARS may allow runners to run longer distances in this type of shoe compared to CRS.

## Introduction

Since the introduction of the Nike Vaporfly 4% running shoe in 2017, considerable improvements in long distance running performance have been observed. The novel shoe technologies combine thick and resilient—but light and cushioning—foam with high longitudinal bending stiffness through a curved carbon fibre plate and have become the new standard for most high-level runners. These shoes are often referred to as “Supershoes” or technologically advanced running shoes (TARS) as compared to classical running shoes (CRS). There is a consensus that TARS enhance running economy and reduce oxygen cost^1–3^, ultimately resulting in improved race times^4,5^. However, decisions on shoe choice are complicated by several factors. Important considerations are the inclusion of only male and predominantly elite participants in previous studies, limited data investigating biomechanical mechanisms for performance improvements, and a high variability of some oxygen uptake and running economy measurements [e.g. differences of − 10 to 13% (expressed as % change)^1^]. Although the introduction of TARS has also resulted in improved performance in females^6,7^ in the field, the influence of the shoes on oxygen cost and biomechanics has not yet been investigated. Further research is needed to establish whether these benefits are observed in other populations, and to understand the mechanisms for such improvements.

The underlying biomechanical mechanisms behind the performance improvements are still unclear^8^. This may be partly due to the fast and recent development of the technology and perhaps due to the narrow focus on a specific group (male elite runners) in most studies performed to date. Few studies have also examined differences in heart rate and the subjective experience (using rate of perceived exertion). In addition, the effect could be distorted by differences in individual effort because participants ran at fixed speeds (e.g. Refs.^2,9^) rather than at a speed that represents equivalent intensity for all participants.

Two mechanisms have been suggested to explain the observed improvements in performance. Firstly, running with TARS may have a lower oxygen cost due in part to a different distribution of work done at the lower extremity joints, such as the ankle and metatarsal phalangeal joint, between shoes^9^. Theoretically, a lower ankle joint moment may be due to smaller forces acting to rotate the ankle or a smaller moment arm to those forces. Alternatively, it is proposed that the carbon fibre plate causes a teeter-totter effect^10^ in TARS which changes biomechanics to reduce metabolic cost during running. Changes in muscle activity affecting joint moments and muscle metabolic cost could be connected to both hypotheses. Yet, both hypotheses are questioned by the observation that cutting the carbon plate did not affect improvements in running economy^11^ leading the authors to suggest that the carbon fibre plate in isolation does not influence ankle biomechanics.

It has been suggested that there is a relationship between running injuries and the biomechanical effects of running shoe design^8,12^, although biomechanical risk factors for running injury remain unclear. For example, a randomised controlled trial in 848 runners showed a greater injury risk when running in hard compared to soft shoes^13^ and a review concluded that, in general, running injury related biomechanical variables vary in different footwear^14^. Case studies of navicular bone stress injuries have been reported in runners who use TARS^15^, but it remains unclear whether this was at least in part influenced by an increase in the training load that it has been anecdotally reported can be tolerated in TARS. Bone stress injuries, such as tibial stress fractures, are a common injury among runners e.g.,^16,17^ and are associated with high magnitudes of tibial loading^18^. There is limited and conflicting evidence about the effect of shoe design on bone loading^19^, and the influence of TARS on bone loading seems particularly important when considering the widespread use of TARS among runners of different levels nowadays.

The aim of the present study was to investigate differences in lower limb joint biomechanics, oxygen uptake, RPE, HR, muscle activation and tibial loading in recreational female and male runners when running with TARS compared to CRS. Tests were performed at individual lactate threshold speed to best represent individual long distance competition speed. We hypothesised lower oxygen consumption when running at individual threshold speed with TARS, while we did not expect to find differences for RPE and HR. Ankle joint plantar flexor moments were expected to be smaller in TARS, as seen in elite runners^9^ and therefore we hypothesised a reduction in tibial bone load in TARS and reduced lower leg muscle activation.

## Methods

### Participants

Nineteen recreational runners, eleven males and eight females (age 27.8 ± 4.2 years; height 174 ± 4.2 cm; body mass 75 ± 14 kg, shoe size EUR39 or EUR43), provided written informed consent to participate in the study. We recruited participants among students and department employees and aimed for even sex distribution. Participants were included if they ran recreationally without following a focussed long-term training schedule and had experience running on a treadmill. Participants were injury-free when data were collected. The protocol was approved by the Ethics Committee of the Norwegian School of Sport Sciences prior to data collection. All experiments were performed in accordance with the Declaration of Helsinki.

### Experimental protocol

Participants visited the laboratory twice with 2–5 days between visits. During the first visit, we determined individual speed at lactate threshold during running, while participants ran at increasing submaximal workloads, separated by 1 min, on the treadmill. The standardised protocol included a 5-min warm-up at self-selected speed and was performed in the participant’s own running shoes.

On the second visit, participants completed a standardised warm-up consisting of two 5-min running workloads at self-selected speed, below their individual lactate threshold, with the CRS (Adidas Adizero Adios 5) and the TARS (Nike Air Zoom Alphafly 1) in random order.

Biomechanical and oxygen uptake data were collected during running in four 5-min workloads where each shoe was worn in random mirrored order (CRS—TARS—TARS—CRS or TARS—CRS—CRS—TARS). We also recorded HR, RPE and spatio-temporal characteristics in each trial to help to describe the shoes. All workloads were performed on a motorised, force-measuring treadmill (M-Gait, Motekforce Link, Amsterdam, The Netherlands) at the participant’s individual lactate threshold speed, as determined during the first laboratory visit. Between trials, participants had 3-min recovery where they changed shoes as required, tying the laces according to what felt comfortable.

### Data collection and analyses

To determine running speed at the individual *lactate threshold*, speed was increased each minute during the submaximal workloads until individual blood lactate values exceeded resting values + 2.1 mmol L^−1^. The speed of the first workload was set based on speed, heart rate and RPE during the warm-up and was increased by 1 km/h for each workload. All participants ran 4–6 workloads before reaching their threshold. Blood lactate was measured after each workload in nonhemolyzed capillary fingertip blood using a standard procedure (Biosen C-line; EKF diagnostics, Cardiff, England) immediately after blood was taken.

Whole body *oxygen uptake* was measured using an automatic ergospirometry system with mixing chamber (Oxycon Pro, Jäger Instrument, Höchberg, Germany^20^). Measurements were averaged over 30 s intervals, and we used the average of the measurements of the last 2 min of each 5-min workload for further analyses.

*Heart rate* was measured continuously using a heart rate monitor (M400, Polar electro OY, Kempele, Finland) and recorded ten seconds before the end of each workload. Participants reported *rate of perceived exertion* (RPE; 6–20^21^) at the end of each workload, which they were verbally and visually reminded of during the last 15 s of each workload.

*Ground reaction force data* were collected from a force-instrumented treadmill (sampling at 1500 Hz) synchronously with motion capture data (Qualisys, Gothenburg, Sweden; sampling at 100 Hz) for a period of 30 s, starting after approximately 4 min into each trial. The 3-dimensional position of 18 reflective markers on the participant’s pelvis and right leg was captured by twelve infrared cameras. To define joint centres and body segments, markers were placed over the right and left anterior and posterior iliac spine, medial and lateral condyles, medial and lateral malleoli, calcaneus, and first, second and fifth metatarsal. Additional tracking markers were placed midway along the thigh and the shank segments on the lateral side of the segments, in rigid clusters of three markers each. Marker trajectories and force data were filtered using a bidirectional second-order Butterworth filter with 15-Hz cut-off. Then, sagittal plane *joint angle, moment and power* for the right knee and ankle joint were calculated using Visual 3D (C-motion, Germantown, MD).

*Tibial bending moments* were calculated in the sagittal plane at the distal third of the tibia in the tibial coordinate system using customised analyses (Matlab R2022a, MathWorks, Natick, MA) as previously described^22–26^. Estimates of the resultant bending moment were based on the sum of external reaction forces and internal muscular forces. The latter were estimated using static optimisation with a cost function that minimised the sum of cubed muscle stresses, constrained to be equal to summed sagittal plane moments of ankle, knee, and hip joint of the right leg^26^. Then, the total of muscular forces and joint reaction forces were applied to the distal tibia. Previous models were used for muscle and joint definitions^27,28^. The eleven muscles spanning the tibial cross section were included in the model (soleus, medial and lateral gastrocnemii, tibialis anterior and posterior, extensor digitorum longus, flexor digitorum longus, flexor hallucis longus, fibularis brevis, fibularis longus, and extensor hallucis longus).

Using the tibial bending moments, we also calculated a *cumulative weighted impulse* to account for the unequal influence of magnitude of bone loading and number of loading cycles on damage accumulation^18,29^. We used the equation by Firminger and Edwards^29^ for cumulative damage calculation:

$$Cumulative\, weighted\, impulse ={\left[n{\int }_{{t}_{i}}^{{t}_{f}}{\left({x}_{s}\right)}^{b} dt\right]}^{1/b}$$

where n is the number of right foot contacts, t_i_ = beginning of stance, t_f_ = end of stance, x_s_ is the measure of internal bone loading (i.e. bending moment time series), b is the tissue-dependent weighting factor that represents the power function between fatigue life and applied stress/strain from experimental data. To account for differences in step frequency between the shoes and to be able to compare damage caused by a certain running distance covered, we also calculated weighted impulse per kilometre for each participant similar to^30^.

*Muscle activity* of soleus and gastrocnemius medialis muscles was measured using a wireless surface electromyography (EMG) system (Myon Aktos, Cometa, Italy), which was integrated to and recorded in Qualisys track manager at 2000 Hz. After shaving and cleaning the recording sites, surface electrodes were placed on the muscle belly of soleus and gastrocnemius medialis according to SENIAM guidelines^31^. All EMG data were rectified and filtered using a fourth order bidirectional Butterworth filter with 8 Hz cut-off frequency. Then, data were normalised for each muscle to each participant’s average peak value of the processed signal from the first two conditions. The first two conditions were chosen to represent both shoes and avoid the influence of fatigue. Integrated EMG was calculated during stance for each muscle and participant by obtaining the area under the EMG—time curve using trapezoidal integration with the central difference method. EMG data from one participant had to be excluded due to issues with the recording of the signal.

### Mechanical shoe testing

**Figure 1:**
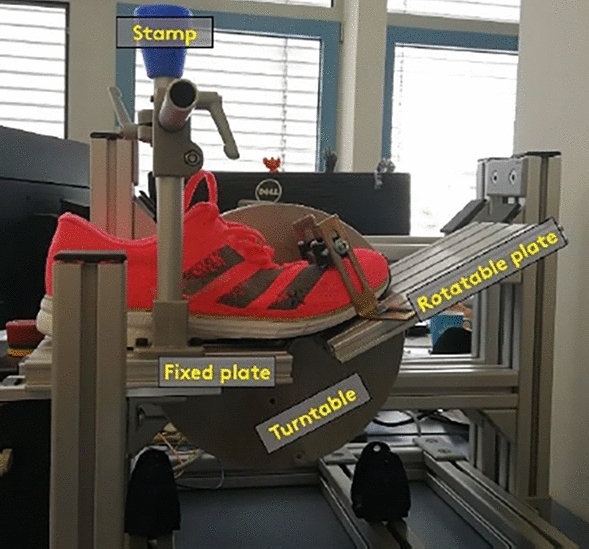
Test bench with a fixed plate and a rotatable plate to test bending stiffness of a shoe (here CRS model) secured with a stamp.

To test bending stiffness of the shoes, an electric test bench (UAS Münster, Germany) was used (Fig. 1). The testing device consists of two rigid plates, one fixed plate and one plate that can be rotated by an electrically driven turntable. The back of the shoe is secured with a stamp on the fixed plate. The flexible plate is pre-rotated to the toe lift, which defines the zero point for determining the bending stiffness. From this starting position, the flexible plate rotates a further 10° in the direction of dorsal flexion for each shoe and torque is determined in this position. The test is repeated five times for each of the four shoes (i.e., both sizes of the TARS and the CRS). Mean and standard deviation were determined for each shoe from all five measurements.

### Data processing and statistics

We averaged the data from the first and second trial for each shoe and each participant to account for unlikely but potential fatigue and habituation effects in our analyses. For continuous data, we used the mean value of ten steps for each participant, shoe, and trial. All data were visually inspected to exclude steps with erroneous data due to e.g., unrecognised markers falling off. The same steps were used for all analyses and variables. Time-series data were normalised to 101 data points for the stance phase by linear interpolation (i.e., EMG, kinematic and kinetic data). For this purpose, stance was determined from heel strike to toe-off of the right foot with a threshold of 25 N. Joint angles are reported relative to the static angle during upright standing with each shoe.

We calculated descriptive statistics for the discrete variables (physiological variables and for cumulative weighted tibial impulse) and compared data between CRS and TARS using two-sided, paired t-tests. Normal distribution was assessed using the Kolmogorov–Smirnov test. Differences in continuous variables (joint kinematics and kinetics, and tibial load) during the stance phase were tested with one-dimensional statistical parametric mapping using two-sided, paired t-tests (available at www.spm1d.org/;^32^). For all statistical tests, the alpha-level was set to 0.05.

## Results

**Figure 2:**
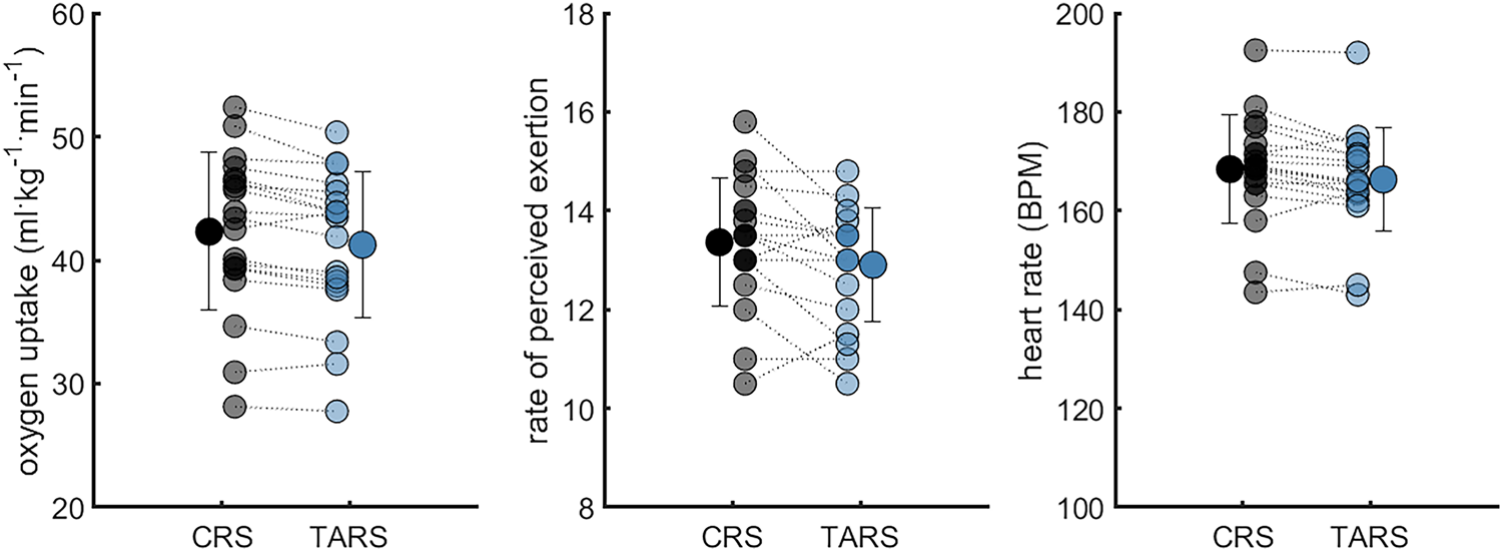
Individual data for oxygen uptake, rate of perceived exertion, and heart rate while running in a classical running shoe (CRS—Adidas Adios) or a technologically advanced running shoe (TARS—Nike Alphafly). Mean and standard deviation are presented as offset points. n = 19.

Participants had 3.0 ± 1.5% lower oxygen uptake with TARS than with CRS while they ran at 12.4 ± 1.9 km/h. RPE and heart rate were 5.0 ± 5.2% and 2.0 ± 0.6% lower in TARS than in CRS (Table 1, Fig. 2).

**Table 1:** Mean and standard deviation (mean (std)) for oxygen (O_2_) uptake, rate of perceived exertion (RPE), heart rate and step cycle and stance duration while running in a classical running shoe (CRS—Adidas Adios) or a technologically advanced running shoe (TARS—Nike Alphafly). Data were compared using paired t-tests (with the alpha level at 0.05). n = 19.

	CRS	TARS	*P*-value
O_2_-uptake (ml kg^−1^ min^−1^)	42.3 ± 6.4	41.2 ± 5.9	0.0003
RPE (6–20)	13.4 ± 1.3	12.9 ± 1.2	0.0259
Heart rate (beats min^−1^)	168 ± 11	166 ± 10	0.0112
Step cycle duration (s)	0.705 ± 0.048	0.714 ± 0.048	< 0.0001
Stance duration (s)	0.335 ± 0.023	0.338 ± 0.023	< 0.0001

Cycle duration was 1.3 ± 0.7% longer in TARS than in CRS and step duration was 1.4 ± 0.6% longer in TARS (Table 1).

**Figure 3:**
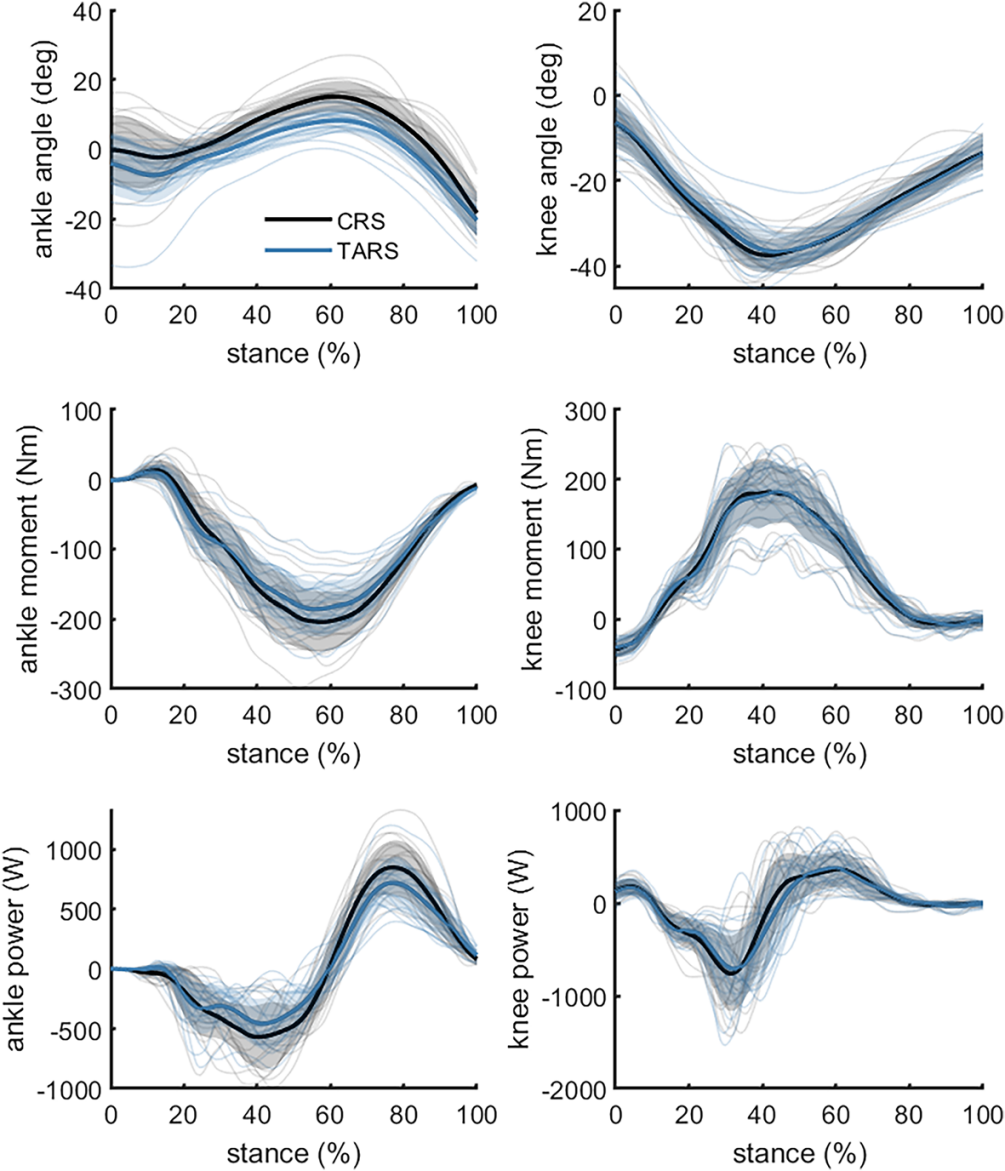
Mean, standard deviation, and individual data for ankle and knee joint angles, joint moment and joint power while running in a classical running shoe (CRS—Adidas Adios) or a technologically advanced running shoe (TARS—Nike Alphafly). Joint angles were calculated relative to upright standing during a static trial with each shoe. n = 19.

Ankle and knee joint angle, moment and power differed in some phases of stance (Fig. 3). The ankle joint was more plantar flexed during stance in the TARS than in the CRS between 2 and 91% of stance (*P* < 0.001). Ankle joint moment and power were lower in the TARS between 42 and 83% of stance (*P* < 0.001) and between 8–14, 29–54, 64–90% of stance (all *P* < 0.001), respectively. Ankle joint work was not different between shoes. Knee joint angle and moment did not differ between shoes (all *P* > 0.05), whereas knee joint power was lower in TARS during a short interval before mid-stance (40–43% of stance; *P* = 0.011).

**Figure 4:**
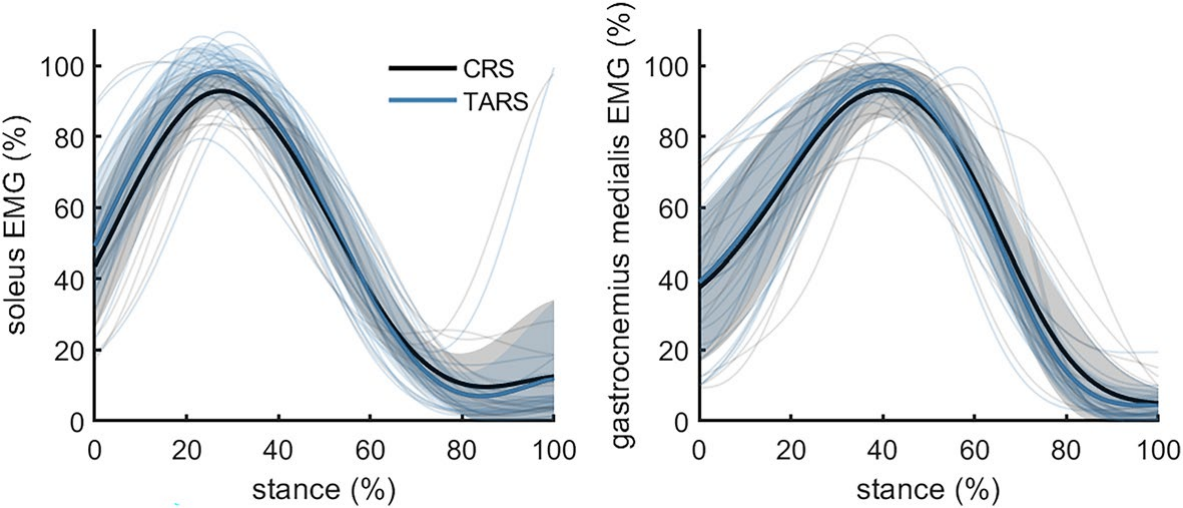
Mean, standard deviation, and individual EMG data for soleus and gastrocnemius muscles while running in a classical running shoe (CRS—Adidas Adios) or a technologically advanced running shoe (TARS—Nike Alphafly). n = 18.

Continuous analyses of soleus and gastrocnemius EMG did not show statistical differences between shoes (Fig. 4). Integrated EMG was not different between CRS and TARS for soleus (16.53 ± 1.90%*s vs. 17.27 ± 2.33%*s; *P* = 0.1104) or for gastrocnemius medialis (18.16 ± 3.15%*s vs. 18.29 ± 2.36%*s; *P* = 0.8008).

**Figure 5:**
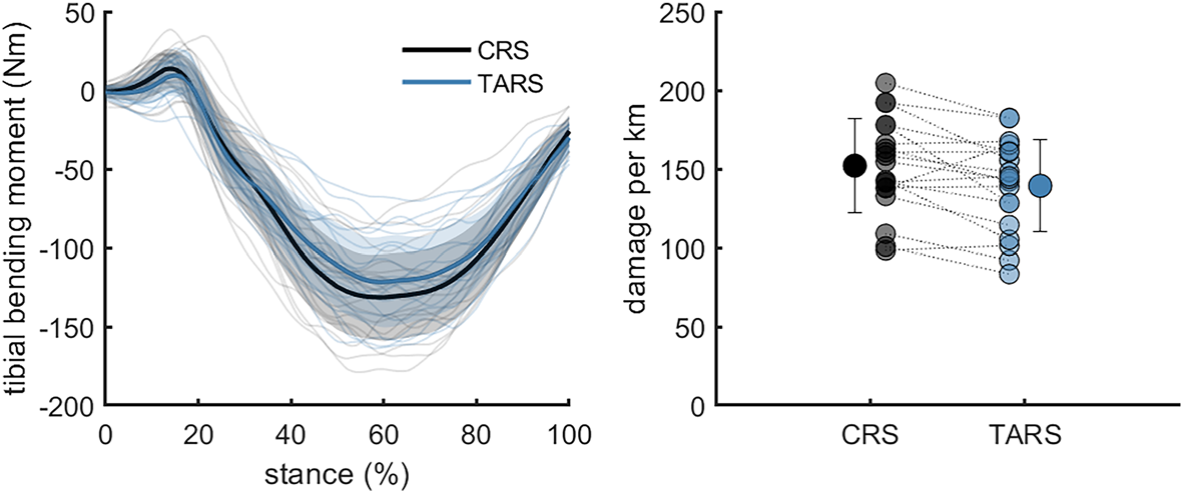
Mean, standard deviation, and individual data for tibial bending moment and tibial damage per kilometre (measured in (Nm^6.6^ s km^−1^)^1/6.6^) while running in a classical running shoe (CRS—Adidas Adios) or a technologically advanced running shoe (TARS—Nike Alphafly). n = 19.

Tibial bending moments were not significantly different between shoes when comparing continuous data (Fig. 5). Cumulative tibial damage per kilometre was 12 ± 9% lower in TARS (139.3 ± 29.2 (Nm^6.6^ s km^−1^)^1/6.6^) compared to CRS (152.1 ± 30.0 (Nm^6.6^ s km^−1^)^1/6.6^; *P* = 0.0051; Fig. 5).

Longitudinal bending stiffness was higher for TARS compared to CRS for both size EUR43 (0.90 ± 0.07 Nm/° vs 0.43 ± 0.03 Nm/°) and size EUR39 (0.86 ± 0.05 Nm/° vs 0.50 ± 0.07 Nm/°).

## Discussion

In this study, we show differences in oxygen uptake, HR, RPE, joint biomechanics, and cumulative tibial load between CRS and TARS during treadmill running among recreational runners. Our hypothesis of lower oxygen consumption when running with TARS was confirmed and the reduction was accompanied by a lower ankle joint moment and power. Tibial loading over the normalised stance phase did not differ between shoes, whereas cumulative loading per kilometre was lower in TARS than in CRS.

The reduction in metabolic cost in our cohort of male and female recreational runners tested at individual threshold speed in the two selected shoe models was consistent with previous studies of male runners^1–3^. This further supports previous findings that running in TARS can benefit running performance at submaximal speeds. The magnitude of differences indicate that the metabolic savings may on average be smaller in recreational than in elite male runners. This difference could be due to the absolute lower running speed^33^ or could be related to individual running intensity. Notably, only two of our nineteen participants had higher oxygen uptake with TARS, one male and one female. Hébert-Losier et al.^1^ also tested a group of recreational runners and reported higher inter individual differences in oxygen uptake of – 10 to 13% between shoes, while we had a variability of − 3 to 6% in our study. A possible explanation for high variability may be the difference in “control” shoe in their study, which was the participant’s own shoe and might therefore have varied significantly in properties. In addition, the choice to use only one measurement of a single trial, which was taken between 2 and 3 min of a workload, may be a less stable measurement method compared to our approach with four measurements.

One previous study reported RPE when comparing different CRS and TARS. They found no differences between shoes for the two lower running speeds tested^1^. However, our findings of on average 0.5 points lower RPE in TARS support these previous findings at higher intensities where RPE was significantly lower with TARS when participants were running at 14.7 km/h (compared with 12.4 km/h in the present study). Although RPE may be biased by participants’ subjectivity (e.g. expectations or design features), the results are in line with the observation that RPE has high validity close to threshold during steady-state activity^34^. Notably, the two participants who did not have reduced metabolic cost with TARS in our study rated RPE as similar and greater in TARS. However, a placebo/psychological effect could play a role in the RPE ratings, firstly, because expectations could have been affected by media reporting of TARS, and, secondly, because we did not control for whether participants had used TARS before. A study design in which participants are blinded to the footwear condition would help to understand whether a placebo effect occurs.

We found differences in joint mechanics similar to previous work, despite this earlier work examining a smaller sample of elite runners at faster speeds using different shoe models^9^. The lack of significant changes in knee joint kinematics and kinetics in both studies rejects the original hypothesis of Hoogkamer et al. (2019) that the compliant midsole of TARS changes the knee’s effective mechanical advantage. However, a smaller ankle joint moment and power in TARS during approximately mid-stance may be due to a smaller resultant moment arm to the ankle joint, as there were no differences in external force at this phase of stance. In turn, this may be due to the thicker foam or the curved, embedded, carbon plate, or the interaction between these two shoe characteristics. The shift towards a more plantar flexed operating joint angle in TARS may also affect the force application point, or rather the centre of pressure under the shoe. Hoogkamer et al. (2019) suggested energy utilisation of the shoe and increased work at the metatarsal phalangeal joint in TARS compensate for the lower requirements at the ankle joint. If so, this may be reflected in muscle activation and contractile behaviour. However, our EMG measurements did not confirm a lower activity of the plantar flexors in TARS, with no differences in the continuous EMG data or the integrated EMG for the biarticular gastrocnemius medialis or for the monoarticular soleus. Another recent study reported lower muscle activity of both gastrocnemii muscles in TARS in athletes^35^. Methodological differences such as protocol, shoe models and characteristics of the participants between the studies could possibly explain the different findings. Alternatively, the normalisation method could have affected the results. Our normalisation could have been affected by fatigue, whereas the normalisation to a maximum isometric contraction used by Hata et al. may be affected by different capacity to contract maximally and different muscle force length properties between participants, which revealed unrealistic activation levels of up to 300% during running. Interestingly, the same study observed no differences in muscle contractile patterns or elastic element behaviour when comparing the shoes. There are to date different hypotheses of how muscle behaviour is affected by changing shoe bending stiffness, e.g., through the addition of carbon fibre plates^36,37^ but no fundamental differences in muscle contractile pattern is expected with different shoe properties.

Our model estimating tibial load showed no significant difference between shoes during each step, but cumulative tibial loading per kilometre was lower in TARS, and this can likely be partly explained by the reduced ankle joint moments in TARS. The lower cumulative loading per kilometre in TARS indicates reduced risk of tibial stress injury development^24^, or that runners can cover on average 12% greater distance with TARS to induce the same cumulative loading on the tibial bone. Estimation of tibial bone load with the current model is particularly important considering the consolidating evidence that measuring external force can be misrepresentative and has only limited or no association with injury risk^38–40^ further supported by our data, which does not show lower ground reaction forces in TARS. A recent study reported navicular bone stress injuries in runners using TARS^15^. However, mechanisms for the development of navicular stress injuries are likely different to those of tibial stress injuries. Furthermore, the findings related to case studies of navicular stress fracture may reflect anecdotal reports of runners increasing their training volume when switching to TARS. Alternatively, the findings of navicular stress injury occurrence in runners using TARS may simply reflect increased attention on TARS leading to the highlighting of these injuries—there was no control group to give an indication of the navicular stress injuries occurring in alternative footwear during the same period. Whilst reduced cumulative weighted impulse of the tibia implies reduced risk of tibial stress injury, there may be increased risk of injury to other tissues. Large prospective studies are needed to determine stress fracture risk in TARS.

High variability in biomechanical and physiological measurements, as well as performance, suggests that individual characteristics of runners may play an important role in the effectiveness of TARS. Therefore, individual analyses have recently been postulated^41^ and should be performed in larger samples in the future. Factors such as foot strike pattern are suggested to interact with TARS^1^, indicating that optimal performance running shoes may require individualised solutions. Further, the influence of fatigue, which alters running kinematics and kinetics^42^, is unknown and it remains difficult to speculate about the effect of long-term tissue adaptations to chronic loading in different running shoes. The relatively good agreement between individual metabolic savings and RPE increases confidence that these results were not due to chance.

An important limitation of our study is that we tested two distinct running shoes with several different characteristics, meaning we cannot distinguish their individual effects. Varying only single parameters has revealed for example that shoe mass increased oxygen uptake by about 1% per 100 g per foot^43^. The effect of mass was small in our study, where the TARS was 14 g lighter for both the men’s and women’s models, according to manufacturer details and had the same weight according to our measurements (232 and 224 g; Balance 500, OBH nordica, Lysaker, Norway). We assume that this small weight difference was not perceived by participants^44^. Another characteristic is longitudinal bending stiffness. The two shoe models in this study showed clear differences in bending stiffness with about double the stiffness in the TARS compared to the CRS. However, a consistent and direct effect of longitudinal bending stiffness on running economy has not yet been identified, possibly due to large individual differences^45^. The influence of other specific characteristics such as carbon fibre plate shape and position, stack-height, drop, or uppers, is still unclear^46^. Notably, isolated effects could disappear or interact differently with other features when combined, further challenging their thorough investigation. It may be insightful to quantify other shoe properties such as energy return. There are limitations induced by testing devices and the properties may interact differently in other environments depending on the testing conditions or rather the individual runner.

A benefit of selecting specific models as in the present study is that they represent a practical scenario and a comparison of two generations of running shoes, i.e., CRS and TARS. Our results may therefore have direct practical implications and help runners, coaches and clinicians when selecting optimal running shoes. Firstly, the lower metabolic savings of TARS in recreational runners compared to elite runners^1,3^ can be relevant to consider from a financial perspective. Furthermore, biomechanical data indicates differences in loading, particularly the reduced cumulative tibial load per kilometre may be relevant for shoe selection and training load management from an injury perspective. The small differences between shoes for RPE and HR may be used as a practical tool for self-assessment of runners who cannot regularly measure oxygen uptake in different shoes or conduct biomechanical analyses.

In conclusion, technologically advanced running shoes reduce the oxygen cost of both female and male recreational runners when running at their individual lactate threshold speed. In addition to these performance benefits, we observed reduced cumulative tibial loading per kilometre. The mechanisms for both reductions may relate to the reduced ankle joint moments when running in these advanced shoes compared with classical running shoes.

## Data Availability

The data supporting the findings of this study are available within the paper. Should any raw data files be needed in another format they are available from the corresponding author upon reasonable request.
